# Thyroid Function with Continuous KRT: A Prospective Study

**DOI:** 10.34067/KID.0000001220

**Published:** 2026-05-04

**Authors:** Matthew Kennis, David Madison, North Foulon, Kayo Okamura, Zhibin He, Isadore Budnick, Mark Yoder, Benjamin R. Griffin, Benjamin J. Kopecky, Melkon DomBourian, John T. Brinton, Samel Park, Warren H. Capell, Sarah Faubel

**Affiliations:** 1 University of Colorado School of Medicine, University of Colorado Anschutz Medical Campus, Aurora, Colorado; 2 Division of Renal Diseases and Hypertension, Department of Medicine, University of Colorado Anschutz Medical Campus, Aurora, Colorado; 3 Division of Pulmonary Sciences and Critical Care Medicine, Department of Medicine, University of Colorado Anschutz Medical Campus, Aurora, Colorado; 4 University of Colorado Hospital, Aurora, Colorado; 5 Division of Nephrology, Department of Medicine, University of Iowa Carver College of Medicine, Iowa City, Iowa; 6 Departments of Medicine-Cardiology and Medicine, University of Colorado Anschutz Medical Campus, Aurora, Colorado; 7 Department of Pathology, University of Colorado Anschutz Medical Campus, Aurora, Colorado; 8 Department of Pediatric Endocrinology, Children's Hospital Colorado, Aurora, Colorado; 9 Departments of Medicine-Endocrinology-Metabolism-Diabetes and Medicine, University of Colorado Anschutz Medical Campus, Aurora, Colorado

**Keywords:** AKI,, dialysis, ESKD, KRT

## Abstract

**Key Points:**

- We identify a novel consequence of continuous KRT: clearance of thyroid-stimulating hormone and thyroid hormone.
- Severe non-thyroidal illness syndrome occurs in most of the continuous KRT patients and is persistent during continuous KRT. Notably, no patient achieved euthyroid status during continuous KRT therapy.

**Background:**

AKI and ESKD requiring continuous KRT (CKRT) are associated with a high mortality rate. Solutes up to 40 kDa in size are amenable to CKRT clearance which includes thyroid-stimulating hormone (TSH; 28 kDa), free thyroxine (fT4; 0.78 kDa), and free triiodothyronine (fT3; 0.68 kDa). The effect of CKRT on TSH and thyroid hormone clearance, thyroid function, and the hypothalamic-pituitary-thyroid axis is unknown.

**Methods:**

This prospective, single-center observational study enrolled 50 intensive care unit patients requiring CKRT and 50 control intensive care unit patients. Serum TSH, fT4, fT3, and reverse triiodothyronine were measured before and on days 1, 3, 8, and 14 after CKRT initiation and at the same time points relative to enrollment in control patients; effluent TSH, fT4, and fT3 were measured on days 1, 3, 8, and 14. Thyroid function status was adjudicated on days 1, 3, 8, and 14. Statistical analyses included employment of linear mixed modeling and time-dependent adjustments, and ANOVA with false discovery rate correction.

**Results:**

Before and during CKRT, CKRT patients had lower fT4 and fT3 levels compared with controls, with a higher proportion of values below the normal reference range. TSH and fT4 were detected in the CKRT effluent, indicating clearance by CKRT. Severe and persistent non-thyroidal illness syndrome (NTIS) was seen in the CKRT cohort. No patient achieved euthyroid status while receiving CKRT.

**Conclusions:**

This is the first study to assess thyroid function and clearance of TSH and thyroid hormones during CKRT. We demonstrate that severe NTIS—characterized by low levels of fT4 and fT3—occurs in most of the CKRT patients. Furthermore, NTIS is persistent in patients receiving CKRT, which may be influenced by CKRT-mediated TSH and fT4 clearance. These novel complications may contribute to the high mortality rate observed in patients with either AKI or ESKD who receive CKRT.

## Visual Abstract

**Figure:**
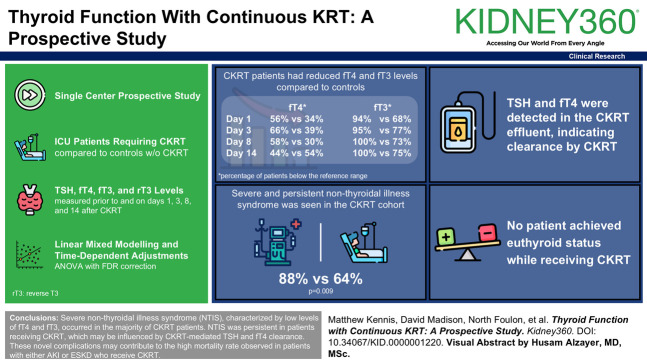

## Introduction

For patients with severe AKI or ESKD requiring KRT in the intensive care unit (ICU), the estimated in-hospital mortality is 50% and 40%, respectively.^1–4^ Solute removal during KRT is indiscriminate and largely dependent on the dialysis membrane size barrier resulting in the clearance of potentially harmful and beneficial solutes alike.^5^ Continuous KRT (CKRT) is commonly used in the ICU, particularly in patients with hemodynamic instability.^6^ Given the uninterrupted nature of CKRT, recipients may be particularly susceptible to the effects of indiscriminate solute removal.^5^ For example, it is well known that CKRT leads to the substantial removal of amino acids, trace elements, vitamins, and certain medications.^7–11^ Whether CKRT removes other potentially beneficial substances including endocrine hormones such as thyroid-stimulating hormone (TSH), thyroxine (T4), and triiodothyronine (T3) is unknown.

TSH, free T4 (fT4), and free T3 (fT3) are low molecular weight hormones, with masses of 28, 0.78, and 0.68 kDa, respectively. Because modern high-flux dialysis membranes used in CKRT permit the filtration of molecules up to 40 kDa,^12^ TSH, fT4, and fT3 are all potentially susceptible to CKRT removal. Currently, however, there is controversy regarding the effect of CKRT on TSH and thyroid hormone clearance with conflicting conclusions in the literature. Case reports have described using CKRT in thyroid storm with associated reduction in serum levels of fT4 and fT3 and have suggested that CKRT could be an adjunct therapy in this condition.^13–15^ By contrast, a recent report of 30 patients receiving CKRT concluded that CKRT did not clear thyroid hormones.^16^ However, these reports only assessed serum levels of thyroid hormones; neither effluent levels nor CKRT clearance were assessed. Thus, while it is plausible that CKRT can clear TSH and thyroid hormones, CKRT clearance of these substances has yet to be investigated.

The removal of TSH and thyroid hormones during CKRT may be problematic for patients in the ICU. Already low levels of fT3 and fT4 are common in critically ill patients as part of non-thyroidal illness syndrome (NTIS). In NTIS, the hypothalamic-pituitary-thyroid (HPT) axis is altered such that, instead of TSH rising in response to reduced thyroid hormone levels (as happens normally with hypothyroidism), TSH remains normal or low. NTIS may affect recovery from critical illness. To date, no study has assessed thyroid function in patients receiving CKRT; thus, the frequency, severity, and course of NTIS in patients with AKI or ESKD who require CKRT is unknown.

We performed a single-center, prospective observational study of CKRT patients and critically ill ICU controls with three objectives: (*1*) determine whether TSH and thyroid hormones are cleared by CKRT; (*2*) assess thyroid function status in CKRT patients before and during CKRT; and (*3*) compare TSH, thyroid hormones, and thyroid function status between CKRT patients and ICU controls. We hypothesized that CKRT-mediated clearance of TSH and thyroid hormones would affect thyroid function, representing a previously unrecognized consequence of this therapy in patients with kidney failure.

## Methods

### Study Population and Design

This prospective, single-center observational study was conducted at the University of Colorado Hospital, a quaternary care center in Aurora, Colorado. Because no prior studies have evaluated CKRT-mediated clearance of TSH or thyroid hormones, no existing data were available to guide enrollment targets. On the basis of our prior experience,^17,18^ we determined that 50 CKRT participants would be sufficient for this pilot study.

Fifty CKRT participants were enrolled between March 2024 and July 2024. Participants were eligible if they were 18 years or older, admitted to an ICU, and receiving CKRT as recommended by the independent nephrology service. Pregnant and incarcerated patients were excluded. Eligible patients or their decision makers were approached within 24 hours of CKRT initiation (day 1). Paired serum and effluent samples of TSH, fT4, fT3, and serum rT3 were collected on days 1, 3, 8, and 14 between 8:00 am and 12:00 pm and were analyzed by the hospital clinical laboratory. TSH, but not fT4, may be affected by diurnal variation,^19^ thus standardized collection times were used. Pre-CKRT measurements used refrigerated serum samples drawn within 24 hours before CKRT initiation (fT3 and rT3 could not be assessed in stored samples). Because our goal was to assess the effect of CKRT, data were collected only while patients were receiving CKRT; missing data therefore arose from patient improvement, discharge, deterioration, or death.

A control cohort of 50 ICU patients not receiving CKRT was enrolled between July 2024 and October 2024. Eligible patients were 18 years or older and receiving ICU care with mechanical ventilation and/or vasopressor or inotropic support, reflecting the clinical profile of most CKRT patients.^20^ Exclusion criteria included pregnancy, incarceration, or receipt of KRT within 5 days. Serum samples were collected on days 1, 3, 8, and 14 between 8:00 am and 12:00 pm; refrigerated pre-enrollment samples were used to assess pre-TSH and pre-fT4 levels. Four control patients initiated CKRT during the study period; data collected after CKRT initiation were excluded. Patients who died or were discharged were excluded from analysis at the point of disposition because still-hospitalized patients were deemed to be the most appropriate control for receiving CKRT.

Study approval was obtained locally from the Colorado Multiple Institutional Review Board, and informed consent was obtained from participants or an authorized representative if the participant was unable to provide informed consent.

### Demographic Data and Definitions

Baseline creatinine and baseline eGFR were determined as previously described.^18^ AKI and stage were classified according to Kidney Disease Improving Global Outcomes serum creatinine criteria: Stage 1 is increase in serum creatinine >0.3 mg/dl from baseline, stage 2 is an increase ≥2× to ≤3× baseline, and stage 3 is an increase ≥3×baseline or a serum creatinine ≥4.0 mg/dl.^21^ Although the need for dialysis is also considered stage 3 AKI, and all CKRT patients would thus meet this definition, we categorized patients on the basis of creatinine criteria to better reflect AKI severity and allow comparison with control patients who did not receive CKRT. ESKD was defined as requiring hemodialysis or peritoneal dialysis for 90 days before enrollment. Sequential organ failure assessment (SOFA) scores were calculated as previously described using the published definition, excluding the renal subscore.^18,22^ Nonsurvivors were patients who died or were transferred to hospice within 30 days of CKRT initiation (CKRT cohort) or study enrollment (control cohort). Patients were classified as Off CKRT if they discontinued CKRT or were discharged before study completion, including one CKRT patient who transferred to another hospital on day 3 and one who declined further blood draws. Control patients were classified as discharged if this occurred before study completion. Demographic data, CKRT parameters, and laboratory values were collected from the electronic health record.

### Measurement of TSH, fT4, fT3, and rT3

Serum and effluent TSH, fT4, and fT3 assays were performed by the University of Colorado Hospital Clinical Laboratory, and serum rT3 assay was performed by ARUP Laboratories in Salt Lake City, Utah. TSH was quantified with a two-site immunoenzymatic assay (Beckman Coulter ACCESS, Loveland, CO). fT4 and fT3 were quantified using a competitive chemiluminescent immunoassay (Siemens ADVIA Centaur, Malvern, PA). rT3 was assessed using quantitative liquid chromatography-tandem mass spectrometry (ARUP Laboratories, Salt Lake City, UT). Serum reference ranges were TSH, 0.45–5.33 mIU/L; fT4, 0.89–1.76 ng/dl; fT3, 2.3–4.2 pg/ml; rT3, 9.0–27.0 ng/dl. Reference ranges for CKRT effluent have not been established.

### Interpretation of Thyroid Function Tests

An endocrinologist (Warren H. Capell), blinded to study cohort and disposition, interpreted thyroid function status using the thyroid function tests (TFTs; *i.e*., TSH, fT4, fT3, and rT3) as summarized in Table 1. The criteria used are similar to those described by Mahashabde *et al*.^23,24^ in that fT3 was interpreted in the context of inappropriate TSH; moreover, our analysis also examined the pattern of HPT axis behavior over the longitudinal sampling period to holistically interpret thyroid function status. Longitudinal assessment is a better approach to categorize patients because trends in the TFTs TSH, fT4, fT3, and rT3 (TFTs) are more reliable than a single point in time. A sensitivity analysis using a higher rT3 threshold (>73.36 versus >36.68) was performed (Table 1). A reproducibility check by WHC after 1 year showed 96% concordance with the original adjudication.

**Table 1:** Adjudication strategy to assess thyroid function status

Thyroid Status	TSH	fT3	fT4	rT3	Other Criteria
NTIS	Variable	↓ or normal	↓ or normal	>36.68 ng/dl	Any of the following
					(*1*) ↓fT3 or ↓fT4+normal/↓TSH, or
					(*2*) Normal fT3/fT4+rT3>36.68+normal/↓TSH, or
					(*3*) ↑TSH (<20)+rT3>36.68 with subsequent downward trend
NTIS recovering	↑ ≥50% from initial NTIS level	Variable	Variable	↓ trend	Must occur after NTIS+↑TSH (≥50%)+↓rT3 trend+TSH must remain <20 mIU/L
Hypothyroidism	↑	↓	↓	<36.68 ng/dl	Must not meet criteria for prior NTIS or NTIS recovering
Subclinical hypothyroidism	↑	Normal	Normal	<36.68 ng/dl	Must not meet criteria for prior NTIS or NTIS recovering
Hyperthyroidism	↓	↑ or normal	↑ or normal	Variable	Must not meet criteria for prior NTIS or NTIS recovering
Subclinical hyperthyroidism	↓	Normal	Normal	Variable	Must not meet criteria for prior NTIS or NTIS recovering
Euthyroid	Normal	Normal	Normal	<36.68 ng/dl	No evidence of NTIS or NTIS recovering at any time point

Thyroid function status was first determined at the Pre/Day 1 time point, with subsequent days interpreted in the context of this baseline status. Day 1 non-thyroidal illness syndrome was defined by: (*1*) ↓free triiodothyronine or ↓free thyroxine and normal/↓thyroid stimulating hormone; or (*2*) normal free triiodothyronine and free thyroxine with normal/↓thyroid stimulating hormone and reverse triiodothyronine >36.68 ng/dl (lower quartile of all reverse triiodothyronine samples); or (*3*) ↑thyroid stimulating hormone (up to <20 mIU/L) and reverse triiodothyronine >36.68 ng/dl with subsequent downward trends. Non-thyroidal illness syndrome recovering was defined by initial evidence of non-thyroidal illness syndrome with a subsequent consistent rise in thyroid stimulating hormone (≥50% increase from initial non-thyroidal illness syndrome level) and further supported by a decreasing trend in reverse triiodothyronine. Once a subject showed evidence of non-thyroidal illness syndrome, they were presumed to have continued non-thyroidal illness syndrome or non-thyroidal illness syndrome recovering; however, a thyroid stimulating hormone rising to >20 mIU/L was considered hypothyroidism rather than non-thyroidal illness syndrome recovering.^23^ Day 1 hypothyroidism was further distinguished from non-thyroidal illness syndrome/non-thyroidal illness syndrome recovering by ↓free triiodothyronine or ↓free thyroxine, ↑thyroid stimulating hormone, and reverse triiodothyronine <36.68 ng/dl, with no evidence of non-thyroidal illness syndrome/non-thyroidal illness syndrome recovering at subsequent time points. Subclinical hypothyroidism was defined as normal free triiodothyronine/free thyroxine, ↑thyroid-stimulating hormone, and reverse triiodothyronine <36.68 ng/dl, with no evidence of non-thyroidal illness syndrome/non-thyroidal illness syndrome recovering at subsequent time points. Hyperthyroidism was defined as ↑free thyroxine or ↑free triiodothyronine and ↓thyroid stimulating hormone, with no evidence of non-thyroidal illness syndrome/non-thyroidal illness syndrome recovering at subsequent time points. Subclinical hyperthyroidism was defined as normal free thyroxine/free triiodothyronine and ↓thyroid stimulating hormone, with no evidence of non-thyroidal illness syndrome/non-thyroidal illness syndrome recovering at subsequent time points. Euthyroid was defined as thyroid-stimulating hormone, free triiodothyronine, and free thyroxine all within the reference limits, with no evidence of non-thyroidal illness syndrome/non-thyroidal illness syndrome recovering at subsequent time points. Non-thyroidal illness syndrome, a syndrome characterized by low levels of free triiodothyronine and/or free thyroxine with an inappropriate central response. Because non-thyroidal illness syndrome going through recovery can have overlapping free thyroxine, free triiodothyronine, and thyroid-stimulating hormone values with hypothyroidism and euthyroid state, a method is needed to distinguish non-thyroidal illness syndrome from these other thyroid states; for this purpose, reverse triiodothyronine > upper quartile was chosen as an indicator of non-thyroidal illness syndrome. This reverse triiodothyronine criterion was needed in 9% of participants. In a sensitivity analysis, we readjudicated thyroid function status and found that a less-conservative criterion (reverse triiodothyronine >73.36) would have changed only 2 of 100 Day 1 classifications. fT3, free triiodothyronine; fT4, free thyroxine; NTIS, non-thyroidal illness syndrome; rT3, reverse triiodothyronine; TSH, thyroid-stimulating hormone.

### CKRT Protocol

CKRT was performed using the NxStage System One S machines and NxStage Purema H filters, which are biocompatible polyethersulfone membranes. Of the 50 CKRT patients, 47 were initiated on continuous venovenous hemodialysis and three on continuous venovenous hemofiltration. CKRT data (time on CKRT, hourly effluent volume, total effluent volume, and average CKRT dose per interval) were obtained from the electronic health record and calculated as previously described.^18^

### Clearance and Sieving Coefficient Calculations

The sieving coefficient (SC) and CKRT clearance were calculated using the following equations as previously described for the clearance of other solutes^25,26^:

$$Marker\;SC=\frac{Effluent\;Marker\;Concentration}{Serum\;Marker\;Concentration}$$

Marker Clearance=Total Effluent Volume×Marker SC

The amount removed by CKRT during each time interval was also calculated using the following equation:

Mass Amount of Marker Removed by CKRT=Total Effluent Volume×Effluent Marker Concentration

To quantify 24-hour removal, we calculated the median (interquartile range) amount removed during the 24 hours preceding days 3, 8, and 14 (excluding day 1 because participants had been on CKRT for only approximately 13 hours).

### Statistical Analysis

Demographic and laboratory parameters were compared between groups using unpaired *t* tests for continuous variables and the Fisher exact test for categorical variables. Day 14 was excluded from some analyses because of substantial attrition.

Owing to laboratory error, seven TSH, four fT4, one fT3, and eight rT3 values were unavailable.

### Between-Group Comparisons at Each Time Point

Linear mixed-effects models (LMM) were used to compare TSH and free T4 between the control and CKRT groups across study days 0, 1, 3, and 8. Models were applied regardless of baseline thyroid function status and to patients with NTIS only. TSH was analyzed after log_2_ transformation to improve residual normality and stabilize variance, whereas free T4 was modeled on the original scale. Study day was treated as a continuous variable, and within-subject correlation was addressed by including subject-specific random intercepts and random slopes for study day. Fixed effects included group (control versus CKRT), study day, and a group-by-study day interaction. Models were adjusted for baseline eGFR, ESKD status, chronic liver disease, and the pre-SOFA score. These covariates were chosen because they differed between groups and are biologically relevant to thyroid function (*e.g*., ESKD status,^27^ chronic liver disease,^28^ and SOFA score^29^). After model fitting, between-group differences at study days pre, 1, 3, and 8 were estimated from model-based least-squares means, with Tukey adjustment for family-wise error. Analyses used *R* (v4.5.1) with lme4, lmerTest, and emmeans packages.

ANOVA with *post hoc* false discovery rate correction for multiple comparisons was used to compare fT3 and rT3 levels for the entire cohort and patients with NTIS for comparisons with more than one time point. LMM was not used because fT3 and rT3 were not determined at the pre time point. For comparisons with only one time point, a Welch *t* test was performed.

## Results

### Control and CKRT Patient Characteristics

As shown in Table 2, the CKRT cohort had a younger median age and higher baseline serum creatinine. Control patients were enrolled median 3 days after hospital admission, and CKRT patients were enrolled a median of 4 days after hospital admission. As expected, the CKRT cohort had patients with more advanced CKD, including ESKD. TFTs in the patients with ESKD relative to last dialysis are reported in Supplemental Table 1. Comorbidities such as hypertension, coronary artery disease, and heart failure were common and similar among both groups, except for chronic liver disease (more prevalent in CKRT patients) and solid malignancies (more prevalent in controls); no patient in either cohort had a history of thyroid malignancy. Rates of hypothyroidism and thyroid hormone supplementation were similar between control and CKRT patients. Use of mechanical ventilation was similar in both groups, while day 1 SOFA score and 30-day mortality were both higher in CKRT patients.

**Table 2:** Control and continuous KRT cohort characteristics

Characteristic, *n*	Control (50)	CKRT (50)	*P* Value
Age, yr, median (IQR)	63 (54–71)	59 (50–69)	0.075
**Sex, no. (%)**			
Male	26 (52%)	31 (62%)	0.31
Female	24 (48%)	19 (38%)	0.31
**Race or ethnic group, no. (%)**			
American Indian or Alaska native	3 (6%)	4 (8%)	>0.99
Black	3 (6%)	7 (14%)	0.32
Hispanic	8 (16%)	10 (20%)	0.80
Other	2 (4%)	1 (2%)	>0.99
White	34 (68%)	28 (56%)	0.30
Baseline serum creatinine, mg/dl, median (IQR)	0.94 (0.74–1.16)	1.07 (0.85–1.39)^a^	0.036
**Baseline eGFR, no. (%)**			
≥60 ml/min per 1.73 m^2^	41 (82%)	27 (54%)	0.005
45–59 ml/min per 1.73 m^2^	3 (6%)	4 (8%)	>0.99
30–44 ml/min per 1.73 m^2^	6 (12%)	5 (10%)	>0.99
15–29 ml/min per 1.73 m^2^	0 (0%)	5 (10%)	0.056
ESKD	0 (0%)	9 (18%)	0.003
**Comorbidities, no. (%)**			
Diabetes mellitus	15 (30%)	13 (26%)	0.82
Hypertension	37 (74%)	32 (64%)	0.39
Coronary artery disease	20 (40%)	24 (48%)	0.555
Heart failure	29 (58%)	26 (52%)	0.69
Chronic liver disease	12 (24%)	23 (46%)	0.035
Chronic obstructive pulmonary disease	14 (28%)	16 (32%)	0.83
Malignancy, hematologic	4 (8%)	7 (14%)	0.52
Malignancy, solid	22 (44%)	6 (12%)	<0.001
Stroke/transient ischemic attack	12 (24%)	15 (30%)	0.65
Hypothyroidism	15 (30%)	9 (18%)	0.24
**ICU service, no. (%)**			
Medical	26 (52%)	26 (52%)	>0.99
Cardiac	6 (12%)	5 (10%)	>0.99
Surgical, cardiothoracic	11 (22%)	6 (12%)	0.29
Surgical, general/trauma	0 (0%)	11 (22%)	<0.001
Neurologic	0 (0%)	2 (4%)	0.49
**Parameters at study enrollment**			
Days in hospital before enrollment; median (IQR)	3 (1–6)	4 (2–10)	0.12
Weight, kg, median (IQR)	85 (68–100)	81 (65–95)	0.39
BUN, mg/dl, median (IQR)	33 (24–50)	55 (33–84)	0.005
Creatinine, mg/dl, median (IQR)	1.65 (1.20–2.40)	3.8 (2.93–5.56)	<0.001
**AKI category, no. (%)**			
No AKI	9 (18%)	1 (2%)	0.016
Stage 1	13 (26%)	8 (16%)	0.32
Stage 2	14 (28%)	7 (14%)	0.14
Stage 3	14 (28%)	25 (50%)	0.04
ESKD	0 (0%)	9 (18%)	0.003
Intubation with mechanical ventilation	29 (58%)	34 (68%)	0.47
**Medication usage, no. (%)**			
Thyroid hormone supplementation	13 (26%)	8 (16%)	0.33
Corticosteroids	30 (60%)	32 (64%)	0.84
Dobutamine	14 (28%)	10 (20%)	0.48
Dopamine	0 (0%)	0 (0%)	>0.99
Day 1 SOFA excluding renal subscore median (IQR)	8 (5–9)	11 (8–13)	<0.001
30-d mortality, no. (%)	13 (26%)	28 (56%)	0.004

CKRT, continuous KRT; ICU, intensive care unit; IQR, interquartile range; SOFA, sequential organ failure assessment.

a *N*=41, no baseline Cr calculated for patients with ESKD.

### TFTs in Controls and CKRT Patients at Study Enrollment (Pre and Day 1)

**Figure 1:**
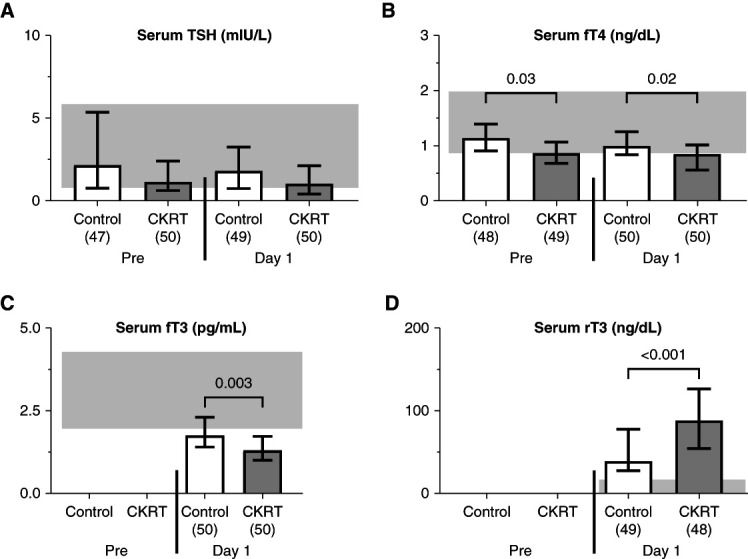
**Serum TSH, fT4, fT3, and rT3 in control versus CKRT patients at the onset of critical illness (pre and day 1).** Serum TSH (A), fT4 (B), fT3 (C), and rT3 (D), from the day before enrollment (pre) and on day 1. To compare TSH and fT4 in control and CKRT groups, a linear mixed model with random intercepts and slopes for time was used, adjusting for baseline eGFR, ESKD status, cirrhosis, and pre-CKRT SOFA score. Intergroup differences at each time point were then assessed using estimated marginal means with Tukey adjustment. To compare fT3 and rT3 in control and CKRT, a Welch *t* test was performed. Only *P* values <0.05 are shown. Data are median and IQR. *N*s are indicated in parentheses. The shaded region represents the normal range for the assay. CKRT, continuous KRT; fT3, free triiodothyronine; fT4, free thyroxine; IQR, interquartile range; rT3, reverse triiodothyronine; SOFA, sequential organ failure assessment; TSH, thyroid-stimulating hormone.

CKRT patients had significantly lower fT4 than controls at the pre and day 1 time points (Figure 1, A–C), significantly lower fT3 than controls on day 1, and significantly higher rT3 than controls on day 1 (Figure 1D).

Laboratory errors affected some samples, so totals may not equal 50 at each time point. In this Figure, and the subsequent Figures and Tables, the number of laboratory tests included in the analyses is reported.

### Thyroid Function Status Over the Course of Critical Illness in Control and CKRT Patients (Pre to Day 14)

**Figure 2:**
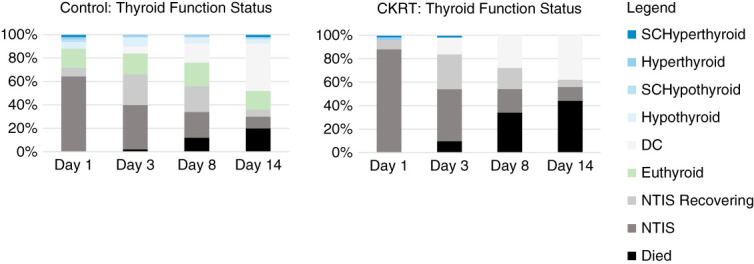
**Thyroid function status and disposition for control and CKRT patients over the course of critical illness (day 1 to day 14).** Thyroid status was adjudicated in a blinded fashion and included the following categories: SCHyperthyroid, Hyperthyroid, SChypothyroid, Hypothyroid, NTIS-recovering, and NTIS. Patients who were DC or died are included in the totals above (if patients were euthyroid before discharge, they were counted as euthyroid if discharged; four control patients went on to CKRT during their illness, and they are included in the discharged category). *N*=50 for all days, for both control and CKRT patients. DC, discharged; NTIS, non-thyroidal illness syndrome; NTIS-recovering, non-thyroidal illness syndrome–recovering; SCHyperthyroid, subclinical hyperthyroidism; SChypothyroid, subclinical hypothyroidism.

Thyroid function assessment for all patients through the 14-day observation period is shown in Figure 2. Figure 2 also includes an accounting of patient disposition (*e.g*., died, discharged). On day 1, CKRT patients were more likely to have NTIS versus controls (CKRT 88% versus controls 64%; *P* = 0.009). None of the CKRT patients were euthyroid at onset or during CKRT.

TFTs over the 14-day observation period relative to the normal reference range is shown in Table 3 and demonstrates that serum fT4 and serum fT3 were both below the normal range in most of the CKRT patients for nearly all time points assessed. Declining numbers reflect attrition from death or discharge, as reported in Figure 2.

**Table 3:** Serum thyroid-stimulating hormone, free thyroxine, and free triiodothyronine values relative to assay reference range

Thyroid Function Paramater	Pre		Day 1		Day 3		Day 8		Day 14	
	Control	CKRT	Control	CKRT	Control	CKRT	Control	CKRT	Control	CKRT
TSH, *n*^a^	47^a^	50^a^	49^a^	50^a^	44^a^	37^a^	30^a^	19^a^	13^a^	9^a^
*N*, (%): <0.45 mIU/L	5 (11%)	5 (10%)	6 (12%)	14 (28%)	7 (16%)	5 (14%)	3 (10%)	1 (5%)	2 (15%)	1 (11%)
*N*, (%): 0.45–5.33 mIU/L^a^	29 (62%)	41 (82%)	34 (69%)	33 (66%)	30 (68%)	28 (76%)	19 (63%)	14 (74%)	6 (46%)	7 (78%)
*N*, (%): >5.33 mIU/L	13 (28%)	4 (8%)	9 (18%)	3 (6%)	7 (16%)	4 (11%)	8 (27%)	4 (21%)	5 (38%)	1 (11%)
TSH median (IQR)	2.15 (0.76–5.36)	1.14 (0.63–2.41)	1.80 (0.75–3.26)	1.03 (0.42–2.12)	1.94 (0.86–3.68)	2.31 (1.08–4.34)	2.95 (1.32–5.85)	2.89 (1.88–4.68)	4.20 (1.4.7–7.33)	1.72 (0.785–3.84)
fT4, *n*^a^	48^a^	49^a^	50^a^	50^a^	44^a^	38^a^	30^a^	19^a^	13^a^	9^a^
*N*, (%): <0.89 ng/dl	9 (19%)	26 (53%)	17 (34%)	28 (56*%)*	17 (39%)	25 (66%)	9 (30%)	11 (58%)	7 (54%)	4 (44%)
*N*, (%): 0.89–1.76 ng/dL^a^	36 (75%)	23 (47%)	31 (62%)	22 (44%)	26 (59%)	13 (34%)	20 (67%)	8 (42%)	6 (46%)	5 (56%)
*N*, (%): >1.76 ng/dl	3 (6%)	0 (0%)	2 (4%)	0 (0%)	1 (2%)	0 (0%)	1 (3%)	0 (0%)	0 (0%)	0 (0%)
fT4 median (IQR)	1.14 (0.91–1.39)	0.86 (0.68–1.07)	0.99 (0.84–1.25)	0.85 (0.56–1.01)	0.97 (0.79–1.20)	0.81 (0.61–0.98)	1.13 (0.84–1.33)	0.83 (0.61–1.09)	0.86 (0.61–1.04)	0.89 (0.65–1.19)
fT3, *n*^a^	—	—	50^a^	50^a^	44^a^	37^a^	30^a^	19^a^	12^a^	9^a^
*N*, (%): <2.3 pg/ml	—	—	34 (68%)	47 (94%)	34 (77%)	35 (95%)	22 (73%)	19 (100%)	9 (75%)	9 (100%)
*N*, (%): 2.3–4.2 pg/ml^a^	—	—	15 (30%)	2 (4%)	9 (20%)	2 (5%)	8 (27%)	0 (0%)	3 (25%)	0 (0%)
*N*, (%): >4.2 pg/ml	—	—	1 (2%)	1 (2%)	1 (2%)	0 (0%)	0 (0%)	0 (0%)	0 (0%)	0 (0%)
fT3 median (IQR)	—	—	1.75 (1.40–2.30)	1.30 (1.00–1.73)	1.90 (1.40–2.20)	1.40 (1.05–1.70)	1.90 (1.60–2.30)	1.60 (1.30–1.80)	1.60 (1.25–2.23)	1.70 (1.45–1.90)

Number (%) of serum thyroid-stimulating hormone, free thyroxine, and free triiodothyronine values below, within, or above the assay reference ranges on pre, day 1, 3, 8, and 14 among continuous KRT and control participants. CKRT, continuous KRT; fT3, free triiodothyronine; fT4, free thyroxine; IQR, interquartile range; TSH, thyroid-stimulating hormone.

a Normal reference range.

### CKRT Parameters

CKRT parameters including dose, time on CKRT, and total effluent volume (for the preceding 24 hours) are reported in Table 4.

**Table 4:** Summary of continuous KRT characteristics and continuous KRT parameters for thyroid-stimulating hormone and free thyroxine

Parameter	Pre CKRT	Day 1	Day 3	Day 8	Day 14
CKRT parameters, total *n*	50	50	42	19	9
Dose, ml/kg per hour	n/a	21.43 (18.35–24.65)	20.4 (17.84–24.25)	17.61 (15.65–20.08)	17.78 (16.17–21.11)​
Time on CKRT, h	n/a	13 (10.25–16.00)	22.5 (20.00–23.00)	22 (17.50–23.00)	24 (23.00–24.00)
Total effluent volume, L	n/a	20.29 (15.23–28.79)	34.72 (28.80–45.09)	29.30 (25.22–37.37)	38.34 (33.55–39.94)​
TSH values during CKRT, total *n*	50	50	37	19	9
Serum mIU/L	1.14 (0.63–2.41)	1.03 (0.42–2.12)	2.31 (1.07–4.34)	2.89 (1.88–4.68)	1.72 (0.79–3.84)
Effluent mIU/L	n/a	0.12 (0.06–0.36)	0.28 (0.14–0.71)	0.42 (0.22–0.99)	0.28 (0.13–0.62)
SC	n/a	0.15 (0.10–0.19)	0.15 (0.12–0.20)	0.16 (0.14–0.21)	0.16 (0.13–0.21)
CKRT clearance, L/24 h	n/a	3.10 (1.69–3.95)	5.94 (4.29–8.07)	5.48 (4.89–6.36)	6.50 (4.73–7.16)
CKRT amount removed, mIU/24 h	n/a	2.54 (1.06–5.51)	11.8 (4.75–24.50)	12.7 (8.97–30.40)	11.5 (4.15–24.00)
fT4 values during CKRT, total *n*	49	50	38	19	9
Serum, ng/dl	0.86 (0.68–1.07)	0.85 (0.56–1.01)	0.81 (0.61–0.98)	0.83 (0.61–1.09)	0.89 (0.65–1.19)
Effluent, ng/dl	n/a	Detected	Detected	Detected	Detected

All parameters are reported as median (interquartile range). Dose, time, effluent volume, clearance, and amount removed per day are as follows: Day 1 is from the initiation of continuous KRT to 8 am the following day, Day 3 is from 9 am. On Day 2 to 8 am on Day 3, Day 8 is from 9 am on Day 7 to 8 am on Day 8, Day 14 is from 9 am on Day 13 to 8 am on Day 14. Serum, effluent, and sieving coefficient are the values obtained from 8 am to 12 pm of the day indicated. Sieving coefficient is the effluent level divided by the serum level; continuous KRT clearance is the total effluent volume×sieving coefficient; continuous KRT amount removed is the total effluent volume×effluent concentration. CKRT, continuous KRT; fT4, free thyroxine; SC, sieving coefficient; TSH, thyroid-stimulating hormone.

### Effluent Levels of TSH, fT4, and fT3

To determine whether removal of TSH and thyroid hormones occurred during CKRT, effluent levels were assessed. Effluent is not an FDA-approved sample type for the TSH, fT4, and fT3 assays used by our hospital laboratory. Therefore, any interfering effect from effluent matrix on measurement of these analytes is unknown. Therefore, to assess the accuracy of effluent measurements, effluent sampled from a mock CKRT circuit (*i.e*., not connected to a patient) was analyzed; samples analyzed included effluent collected directly from the dialysate bag, and then prefilter and postfilter at 5, 20, and 25 minutes (*n*=7 samples total). These blank effluent samples were assessed for TSH, fT4, and fT3 using the same clinical platform as serum. In all samples, TSH levels were below the detection limit (*i.e*., <0.01 mIU/L); for fT4, levels were 0.12±0.03 ng/dl; for fT3, levels were 2.24±0.03 (mean±SD). Then, to quantify potential imprecision introduced by effluent matrix a within-run replicate study was performed using a Clinical Laboratory and Standards Laboratory Institute protocol commonly used in the clinical laboratory setting.^30^ Effluent from the postfilter 25-minute sample was measured ten consecutive times for TSH, fT4, and fT3. Replicate measurements for TSH were all undetectable. fT4 was 0.178±0.048 ng/dl with a coefficient of variation percentage of 27%. fT3 was 2.13±0.28 pg/ml with a coefficient of variation percentage of 13%.

Together, these data indicate that there was no matrix interference with the TSH assay and that these measurements can be considered reliable. For fT4, most of the values were greater than the mean of the blank effluent measurements and the replicant study (*i.e*., 92% were >0.12; 52% were >0.178; *n*=109) indicating that fT4 is detectable in the effluent; however, the imprecision introduced by matrix interference indicates that levels cannot be accurately quantified. For fT3, the mean values in the blank effluent (2.24) and replicant study (2.1) were greater than all of the effluent fT3 measurements from patients (1.26±0.29 pg/ml, *n*=111) indicating substantial matrix interference for the assay and an inability to determine the presence or absence of fT3 in the effluent.

### SC, CKRT Clearance, and Daily Removal of TSH

The SC of TSH was 0.15, 0.15, 0.16, and 0.16 on days 1, 3, 8, and 14, respectively (Table 4), indicating that approximately 15% of serum TSH is continuously removed during CKRT, equating to the removal of 12 mIU per 24 hours of CKRT.

### TSH and Thyroid Hormones in Control and CKRT with NTIS

**Figure 3:**
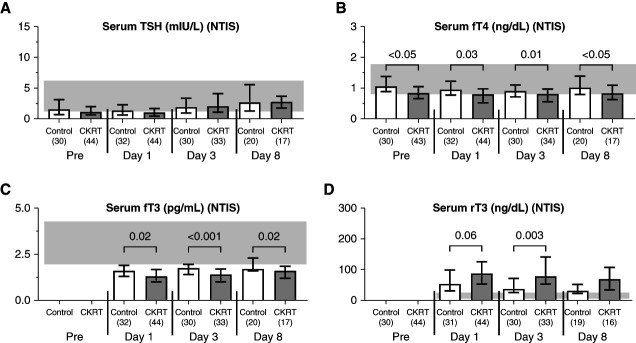
**Serum TSH, fT4, fT3, and rT3 in control and CKRT patients with NTIS.** Serum TSH (A), fT4 (B), fT3 (C), and rT3 (D) in control and CKRT patients with NTIS. To compare TSH and fT4 in control and CKRT groups in patients with NTIS, a linear mixed model with random intercepts and slopes for time was used, adjusting for baseline eGFR, ESKD status, cirrhosis, and pre-CKRT SOFA score. Intergroup differences at each time point were then assessed using estimated marginal means with Tukey adjustment. To compare fT3 and rT3 in control and CKRT, ANOVA with FDR adjustment was performed. Only *P* values <0.05 are shown. Data are median and IQR. *N*s are indicated in parentheses. The shaded region represents the normal range for the assay. FDR, false discovery rate.

Having established that CKRT removes TSH and fT4, we next compared serum levels of TSH, thyroid hormones, and rT3 in CKRT patients and controls. To ensure that the comparison groups had similar baseline HPT axis and function, these analyses were limited to patients with NTIS. As shown in Figure 3, serum fT4 and fT3 were lower at all time points in CKRT versus controls, indicating more severe and persistent NTIS status.

### TSH and Thyroid Hormones in Control and CKRT with NTIS Grouped by SOFA Score

**Figure 4:**
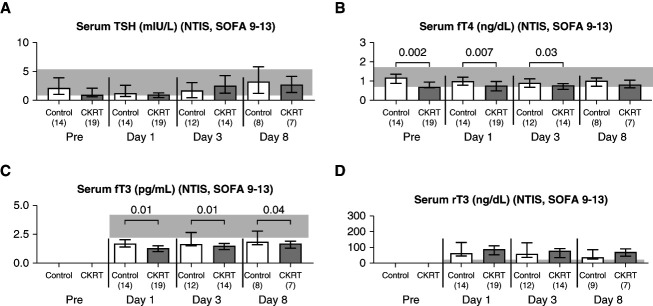
**Serum TSH, fT4, fT3, and rT3 in control and CKRT patients with NTIS and a similar SOFA score.** Serum TSH (A), fT4 (B), fT3 (C), and rT3 (D), in control versus CKRT patients with NTIS and a SOFA score between 9 and 13. Comparisons between control and CKRT were determined by ANOVA with FDR correction. Only *P* values <0.05 are shown. Data are median and IQR. *N*s are indicated in parentheses. The shaded region represents the normal range for the assay.

**Figure 5:**
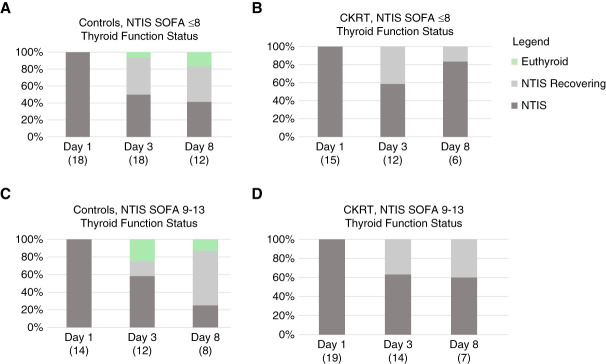
**Thyroid function status in control and CKRT patients with NTIS and a similar SOFA score over the course of critical illness.** Thyroid function status in controls versus CKRT with NTIS and SOFA score ≤8 (A and B) and SOFA score 9–13 (C and D).

Because severity of critical illness is associated with NTIS severity and SOFA scores were higher in CKRT patients, patients were also stratified by SOFA score on the basis of tertiles (*i.e*., SOFA score ≤8, SOFA score 9–13, and SOFA score ≥14)—to directly compare CKRT and control patients with similar severity of critical illness in addition to its inclusion in the LMM analysis described above (of note, no control patient had a SOFA score ≥14). As shown in Figure 4, CKRT patients with NTIS and a SOFA score of 9–13 had significantly lower fT4 and fT3 levels for most of the time points assessed. This difference was not observed in patients with a SOFA score ≤8 (Supplemental Figure 1). TSH and rT3 were similar in both groups. Thus, severe NTIS was present and persisted in CKRT patients compared with controls with a similar severity of illness. Adjudicated thyroid status was also compared in NTIS patients based on SOFA score grouping. As shown in Figure 5, A–D, NTIS was prolonged in participants receiving CKRT, regardless of SOFA score category.

## Discussion

This study addresses the knowledge gap regarding thyroid function before and during CKRT. The major findings are that (*1*) patients requiring CKRT have a greater rate and severity of NTIS compared with other critically ill patients; (*2*) both TSH and fT4 are removed by CKRT; and (*3*) patients receiving CKRT have prolonged NTIS. These novel complications may contribute to the high mortality rate observed in patients with either AKI or ESKD who require CKRT.

Overall, the data in the present report demonstrate that a broad spectrum of thyroid function abnormalities exists in critically ill patients (with or without CKRT requirement), including hypothyroidism, hyperthyroidism, and NTIS. Of the 100 critically ill patients assessed, only eight were euthyroid at study enrollment (all of whom were controls). NTIS was the predominant abnormality in CKRT patients, affecting a significantly greater proportion at onset (88% versus 64% in controls) and persisting throughout the course of CKRT. Notably, no patient achieved euthyroid status while receiving CKRT during the 2 weeks of assessment. Furthermore, NTIS at onset was more severe in CKRT patients with 59% having a fT4 level that was below the reference range; in contrast, only 28% of NTIS controls had lower fT4, similar to rates observed in other populations of critically ill patients.^29,31^ NTIS with low fT4 is a clinically relevant subset of NTIS because it is associated with poor outcomes including longer ICU stays, greater rates of mechanical ventilation, and increased mortality compared with those with normal fT4^29^—findings also observed in patients requiring CKRT.

We demonstrate for the first time that fT4 and TSH are removed by CKRT. It is possible that removal of fT4 and TSH has clinical consequences because the severe NTIS phenotype observed at enrollment persisted during CKRT, with consistently low fT4 and fT3. Recovery from NTIS requires a rise in TSH followed by rising thyroid hormones. However, despite lower fT4 and fT3, TSH was not significantly elevated in CKRT patients versus controls, suggesting these patients cannot adequately stimulate the HPT axis sufficiently (*via* TSH) to restore thyroid hormone levels. We postulate that the added stress from removing both fT4 and TSH by CKRT may contribute to this severe phenotype: fT4 removal may directly affect serum fT4 levels, keeping these levels low; and TSH removal may prevent increased fT4 production because rising TSH is the compensatory stimulus for fT4 production. On the basis of quantities removed, we suspect that TSH clearance may have the greatest clinical effect because the approximately 12 mIU removed per day represents more than 10% of normal daily production and likely a much larger proportion of daily production in critically ill patients, whose TSH synthesis is reduced.^32^

Regardless of the mechanisms driving NTIS severity and duration, our findings are clinically significant for CKRT patients because NTIS with low fT4 and/or fT3 is associated with increased ICU mortality.^29,33–36^ While the adaptive versus maladaptive nature of NTIS early in critical illness remains debated,^37^ prolonged NTIS may exert harmful effects resembling hypothyroidism, including prolonged mechanical ventilation,^31^ cognitive impairment, myocardial dysfunction, muscle weakness,^37^ immune dysfunction,^38^ and fluid retention with generalized edema^39^—all of which may impair recovery from critical illness. Although previous studies of thyroid hormone supplementation in NTIS have shown mixed, but largely negative results for clinical outcomes,^37^ none focused on patients with the added stress of CKRT. Our data suggest that there may be a subset of severely ill CKRT patients (*e.g*., SOFA scores >9) who have an HPT axis that cannot adequately compensate and who might benefit from hormone replacement or thyrotropin-releasing hormone analogs.

This study has several strengths. Unlike many CKRT studies that estimate solute clearance from prescribed dose or modeling, we measured total effluent volume per 24 hours and paired serum and effluent samples over several days, enabling calculation of SC, clearance, and daily TSH removal. Our cohort of 50 CKRT patients is large relative to other CKRT studies, and we included a contemporaneous critically ill control group. TSH, fT4, and fT3 were measured by the clinical laboratory using freshly collected samples at a standardized time. Thyroid status adjudication was rigorous, assessing all TFT parameters longitudinally rather than relying on a single hormone at one time point. Rigorous laboratory methods were used to assess effluent matrix interference. The prospective design over several days is uncommon in both the CKRT and NTIS literature.

This study has several limitations. Despite including a control group, differences between cohorts were noted; statistical adjustment was performed, but residual confounding cannot be excluded. Patient attrition because of our inclusion criteria resulted in missing data inherent to the prospective observational design. While low fT3 and fT4 are predictors of ICU mortality^33–36^ and were lower in CKRT patients, assessing whether fT4 or fT3 are independent mortality risk factors requires a larger, dedicated study. Membrane adsorption was not studied, which may have led to underestimation of hormone clearance. Effluent fT3 could not be quantified and some matrix interference was identified for fT4; quantitative effluent levels of both hormones may be better assessed with different assays. Notably, the two-site immunoenzymatic (sandwich) assay used for TSH did not exhibit the matrix interference seen with the competitive chemiluminescent assay. Finally, medications known to suppress the HPT axis (*e.g*., corticosteroids and dobutamine)^40,41^ were used, although utilization rates were similar between groups.

This is the first study to assess thyroid function and clearance of TSH and thyroid hormones in CKRT patients. We found that TSH and fT4 are removed by CKRT. We demonstrate that severe NTIS—characterized by low levels of fT4 and fT3—occurs in most of the CKRT patients with AKI or ESKD. Furthermore, NTIS is persistent in patients receiving CKRT, which may be influenced by TSH and fT4 clearance. These novel complications may contribute to the high mortality rate observed in patients with either AKI or ESKD who receive CKRT.

## Supplementary Material

## Data Availability

Original data generated for the study will be made available upon reasonable request to the corresponding author. Data Type: Observational Data; Raw Data/Source Data. Reason for Restricted Access: This is low-throughput experimental data; thus original data will be made available upon reasonable request to the corresponding author.

## Declarative Statements

This study includes clinical experimentation and received Institutional Review Board or Ethics Committee approval. All patients provided written informed consent. This study includes clinical experimentation and complies with the Declaration of Helsinki.

## Supplemental Material

Supplemental Table 1. TFTs in patients with ESKD.

Supplemental Figure 1. Serum TSH, fT4, fT3, and rT3 in patients with NTIS and a SOFA score ≤8.
