# Supplementary material for: Thyroid Function with Continuous KRT: A Prospective Study
Source: Kidney360. 2026 May 4;7(7):1522–34. doi: 10.34067/KID.0000001220 (PMC13450954; doi:10.34067/KID.0000001220)
Supplement: Supplementary file 2 [file kidney360-7-1522-s002.pdf]

Patient ID	Last iHD (days)	Thyroid status	Pre		Day 1			
			TSH	fT4	TSH	fT4	fT3	rT3
ESKD 1	-1	NTIS	1.14	1.07	1.33	0.89	1.0	53
ESKD 2	-1	NTIS-Recovering	3.70	0.95	5.30	0.94	0.8	163
ESKD 3	-2	NTIS	0.65	0.46	0.56	0.43	0.8	131
ESKD 4	-2	NTIS	1.89	1.53	1.76	1.27	1.4	79
ESKD 5	-3	NTIS	1.17	0.61	2.62	0.35	0.5	10
ESKD 6	-6	NTIS-Recovering	12.47	1.10	5.60	1.01	1.0	67
ESKD 7	-7	NTIS	7.39	0.70	2.34	0.79	0.9	90
ESKD 8	-7	NTIS	1.62	0.39	1.29	0.28	0.7	38

Supplemental Table 1. Thyroid function tests in ESKD patients. The timing of last KRT (intermittent hemodialysis (iHD) in all cases) relative to the initiation of CKRT and the thyroid function tests is shown in the table. No discernible pattern among TFTs with regard to the recency of hemodialysis is observed, particularly for fT4.

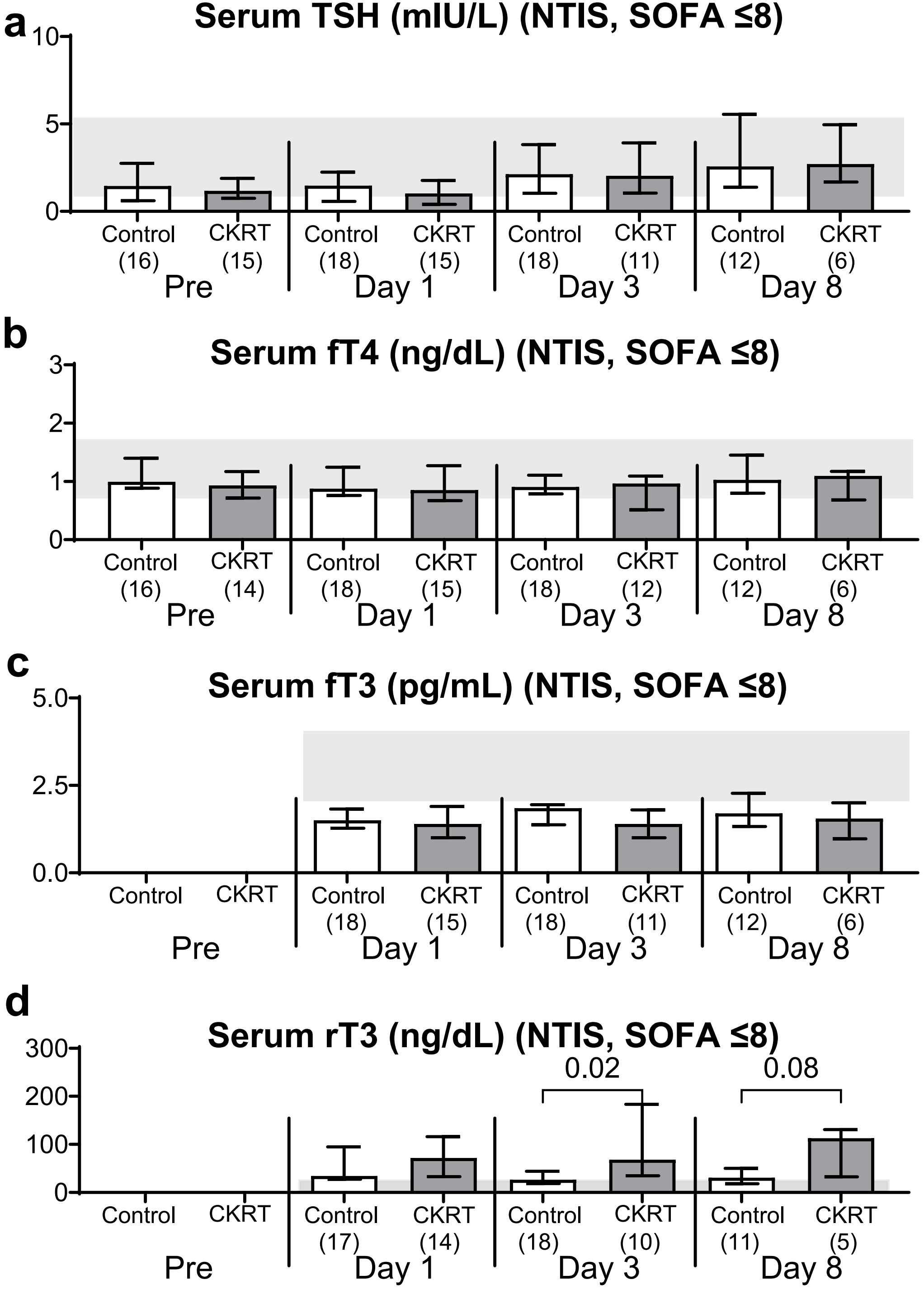

Supplemental Figure 1. Serum thyroid stimulating hormone (TSH), free thyroxine (fT4), free triiodothyronine (fT3), and reverse T3 (rT3) in patients with NTIS and a SOFA score ≤8. Serum TSH (a), fT4 (b), fT3 (c), and rT3 (d) were determined in controls vs. CKRT with NTIS and SOFA score ≤8. Comparisons between control and CKRT were determined by ANOVA with FDR correction. Data are median and IQR. Ns are indicated in parentheses. The shaded region represents the normal range for the assay.
